# Improved Performance in Multilayer Paper Composites Through the Incorporation of Inorganic Nanomaterials into Sodium Silicate Adhesive

**DOI:** 10.3390/ma19091897

**Published:** 2026-05-05

**Authors:** Douglas Lamounier Faria, Julio Soriano, Leticia Catta Preta da Silva, Anand Ramesh Sanadi, Gustavo Henrique Denzin Tonoli

**Affiliations:** 1 Department of Forest Science, Federal University of Lavras—UFLA, Perimetral Av., P.O. Box 3037, Lavras 37200-900, MG, Brazil; gustavotonoli@ufla.br; 2 School of Agricultural Engineering, University of Campinas—UNICAMP, Candido Rondon Av, Campinas 13083-875, SP, Brazil; julio.soriano@feagri.unicamp.br; 3 Tubominas Packaging Industry Ltda., Elói Mendes Av., 281, Industrial District, Elói Mendes 37110-000, MG, Brazil; sgq@tubominas.com.br; 4 Department of Geosciences and Natural Resource Management, Faculty of Science, University of Copenhagen, Rolhedsvej 23, 1958 Frederiksberg, Denmark; anrs@ign.ku.dk

**Keywords:** inorganic fillers, recycled paper, bonding property, bond line quality, thermal analysis

## Abstract

Multilayer paper composites have been widely applied in industrial sectors and as sustainable concrete formwork in civil construction. These materials are produced by pressing paper layers bonded with a sodium silicate adhesive; however, their structural performance is often limited by the adhesive’s low mechanical strength. Therefore, in this study, the effects of incorporating 0.5 wt.% nanoclay (NA), nanosilica (NS), and kaolin into sodium silicate on the physical, mechanical, and microstructural properties of the composites were evaluated. The composites were fabricated with 20 layers of recycled kraft paper and a final mass of 65 g/m^2^ of reinforced sodium silicate applied by a glue line. The adhesive was applied using a paper coating machine, followed by pressing at 4.30 MPa. The results showed that the presence of nanomaterials had no measurable effect on the apparent density of the composites; nevertheless, the inclusion of 0.5% NA promoted a 25% increase in toughness. Thus, the use of nanomaterials is efficient at obtaining better-quality composites for numerous technological applications.

## 1. Introduction

Cardboard tubes are materials formed by multiple layers of kraft paper bonded by adhesive through a process of spiral winding of the paper layers, making paper tubes ideal for different technological applications, such as in the packaging, textile, food, pyrotechnic, polymer, paper production, and construction industries, where these tubes can be used as molds for reinforced concrete columns [1]. The traditional method of producing these tubes involves pulling the paper layers at a specific angle onto a cylindrical shaft, which defines the internal diameter of the paper tube. The total diameter of the tubes depends on the number of glued paper layers, with the adhesive layer bonding these layers generally being negligible. As it is a continuous process, the length of the tubes depends on the factory’s production size or logistical limitations [1].

Different types of adhesives, such as starch, polyvinyl acetate (PVAc/PVA), polyurethane, acrylic, hot melt, dextrin, natural latex, and synthetic rubber, have been used in the production of cardboard tubes [2,3]. Some types of organic materials, such as phenolic compounds and epoxy, have been employed because of their exceptional physicochemical properties, which promote satisfactory adhesion strength. However, these organic adhesives present some challenges related to their use, such as brittleness in situations with thermal load or aggressive environments and the release of volatile organic compounds (VOCs) and formaldehyde, which are associated with environmental pollution and human health problems because of their carcinogenic action [4]. An environmentally friendly alternative to overcome these inherent challenges in the production of cardboard tubes is the use of sodium silicate as an adhesive. This adhesive has interesting functional properties that make it promising for bonding multiple layers of paper; it is an inorganic adhesive that is low cost and easily obtainable and has exceptional thermal stability, good adhesion, and sustainable development potential [5,6]. However, compared with organic adhesives, sodium silicate has low adhesion strength and is more sensitive to moisture [7]. The selection and application of the adhesive are critical to ensuring the long-term performance and reliability of the lamination process. Accordingly, this procedure must be carefully controlled, considering the characteristics of the substrates and the specific processing conditions involved [8].

The technological quality of multilaminated composites is governed by a series of factors, such as the pressing pressure [9], number of pressing cycles [10], type of binder used [11,12,13], basis weight and number of adhesive layers [14], and presence of additives in the adhesive [15]. The modification of sodium silicate adhesives with organic and inorganic additives significantly improves the properties of the composite, such as increasing the shear strength of the glue line, reducing the zones with stress concentration, increasing the mechanical durability and dimensional and thermal stability, and in turn reducing the void content [16,17,18,19].

Nanomaterials exhibit unique nanoscale properties, making them promising candidates for enhancing the performance of adhesives used in the bonding of paper layers. The high aspect ratio and large surface area of the nanoclay particles significantly increase the interface area with the adhesive and favor efficient stress transfer, with a significant improvement in the mechanical properties of the composite [20,21]; the exceptional thermal resistance of nanosilica hinders the diffusion of heat and decomposition products, acting as a physical barrier, in addition to filling with microvoids and defects, improving fracture resistance [22]. In addition to being natural, abundant, low-cost, and generally nontoxic, kaolin is a nanomaterial with properties of interest for use in composites, constituting an environmentally friendly alternative to synthetic fillers or more expensive nanoreinforcements [23]. The incorporation of nanomaterials as additives in adhesive systems provides several potential benefits, including enhanced mechanical performance, improved durability, and greater resistance to environmental degradation [8].

The incorporation of nanomaterials as additives in adhesive formulations can lead to significant performance enhancements by modifying the adhesion and cohesion properties of the adhesive during the lamination process. The application of nanomaterials in wood science and technology is attracting significant interest for enhancing the properties of wood-based composite materials [24]. Faria et al. [6] investigated the influence of cellulose nanofibrils (CNFs) on the physical, mechanical, thermal, and rheological properties of sodium silicate adhesive. Additionally, the authors evaluated the combined effect of the nanoreinforced adhesive and longitudinal cuts along the kraft paper in multilaminated paper composites. On the basis of static bending tests, the authors reported that the insertion of 0.5% CNFs as a reinforcement to sodium silicate resulted in a significant increase of 20.4%. In a study on laminated wood composites glued with polyvinyl acetate (PVAc), urea-formaldehyde (UF), and epoxy (EPX) adhesives, Pelit et al. [8] reported that the incorporation of different amounts of nanomaterials (ranging from 0.25 to 2%) promoted a significant increase in the mechanical strength of the evaluated composites. For the composites with *Poplar wood*, a significant 12% increase in the shear strength of the glue line was observed for the adhesive reinforced with 1% NS. Çakir and Kinay [25] investigated the effects of NS, NA, and multiwalled carbon nanotubes at varying concentrations (0.5%, 1%, 1.5%, and 2% wt.%) on the mechanical properties of EPX adhesive. The authors reported that the insertion of inorganic nanoreinforcements significantly increased the shear strength of glued wood joints, where reinforcements with 0.5 wt.% NS and 0.5 wt.% NA increased the strength by 20.1% and 25.6%, respectively. Dinesh et al. [26] reported that the incorporation of NS as a reinforcement in EPX adhesive in proportions ranging from 0.5% to 2% (wt.%) was able to increase the bending strength by 25% with 0.5% incorporation, whereas the maximum content (2%) resulted in a 37.5% increase. Liu et al. [17] reported that the insertion of magnesium oxide and NS in a sodium silicate adhesive led to a 74% increase in the bending strength of composites produced with NS-to-magnesium oxide ratios of 1:1. Recently, Furtini et al. [27] evaluated the influence of varying concentrations of NS (0, 1 and 2% wt.%) on the physicochemical, rheological, and thermal properties of cardanol-formaldehyde adhesive for the production of wood particle composites. According to the authors, the insertion of 2% NS led to agglomeration in contact with the adhesive, with the viscosity increasing from 22 to 346 Pa·s. However, the thermal stability of the adhesive nanoreinforced with 1% NS was close to that of the pure adhesive, indicating that high levels of inorganic reinforcement reduce the maximum degradation temperature of the adhesive system.

This study addressed the gaps concerning the effects of incorporating clay, nanosilica, and kaolin nanoparticles on the physical, mechanical, and microstructural properties of multilayer paper composites bonded with a sodium silicate adhesive. On the basis of the methodology and results of Faria et al. [6], this research aimed to advance the development of laminated composite materials obtained from adhesive systems reinforced with nanomaterials. The incorporation of these inorganic nanomaterials as reinforcing agents in the sodium silicate matrix represents an innovative strategy to improve composite performance, offering a sustainable alternative for the production of multilayer paper composites with potential applications in various industrial sectors.

## 2. Materials and Methods

### 2.1. Materials

**Figure 1:**
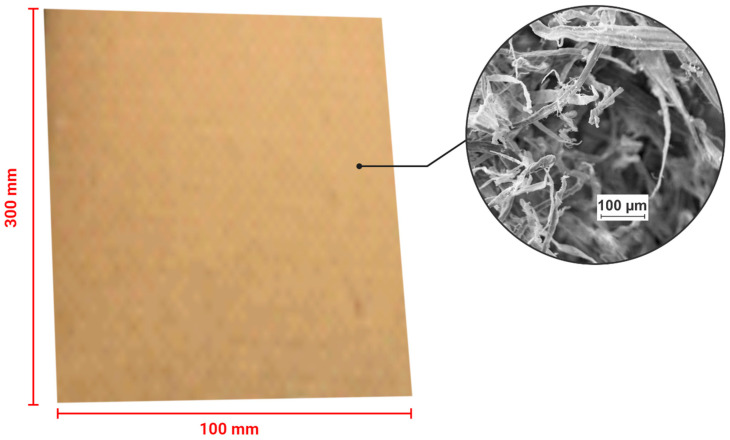
Details of the kraft paper used in the production of the composites.

Composites were produced from 20 layers of recycled kraft paper (grammage ~182 g/m^2^), cut to dimensions of 100 mm × 300 mm (width × length) (Figure 1). The paper reels were processed using a cutting machine prior to lamination. A sodium silicate adhesive with semialkaline character (pH 11.46) and ~49% solids content was used for adhesion. The adhesive properties are presented in Table 1.

The nanomaterials nanoclay (NA), nanosilica (NS), and kaolin were used as supplied by the manufacturers. The nanosilica used in the present work, which was 99.8% pure and had a particle size between 10 and 20 nm, was obtained from HW Nanoparticles (Guangzhou, China). The kaolin used, formula Al_2_Si_2_O_5_(OH)_4_, was obtained from Exodus Científica (Sumaré, São Paulo, Brazil). Commercial Cloisite^®^ Na+ montmorillonite nanoclay supplied by Southern Clay Products, Inc., was used (Louisville, KY, USA); it is a natural sodium montmorillonite that has an apparent density of 0.33 g/cm^3^ and an initial basal spacing of 1.17 nm.

### 2.2. Characterization of Kraft Paper and Nanomaterials

FTIR spectra of the kraft paper and nanomaterials were obtained with a Nicolet 470 Nexus spectrometer (Nexus, Chicago, IL, USA) in transmission mode. The system was continuously purged with nitrogen, and each spectrum was collected from 4000 to 400 cm^−1^ using 64 scans at a spectral resolution of 2 cm^−1^.

The microstructure of the kraft paper and nanomaterials was analyzed by field emission scanning electron microscopy (SEM-FEG). The samples were coated with a gold layer using an evaporator and subsequently observed with a TESCAN CLARA ultrahigh-resolution SEM (Libušín, Czech Republic) at 10 kV.

### 2.3. Thermogravimetry (TGA), Rheology, Wettability, and Water Absorption of the Nanoreinforced Sodium Silicate Adhesive (Cobb Test)

The thermal degradation of the nanoreinforced adhesive was evaluated using a DTG Shimadzu 60 thermogravimetric analyzer (Kyoto, Japan). An adhesive sample (~10 mg), nanoreinforced with 0.5% NA, 0.5% NS, and 0.5% kaolin, was prepared in powder form by drying at 105 ± 3 °C for 24 h and subsequent ball milling. The sample was then placed in the holder and heated to 600 °C at a heating rate of 10 °C/min under a nitrogen atmosphere (60 mL/min).

Rheological behavior is a key factor in composite fabrication, as it directly influences adhesive spreading on the paper substrate. The nanoreinforced sodium silicate adhesive was analyzed using an Anton Paar MCR 102e rheometer (Anton Paar GmbH, Graz, Austria) fitted with a 20 mm parallel plate geometry to examine its structural and flow characteristics under applied shear. Measurements were conducted at 25 °C across a shear rate interval of 0.1–100 s^−1^, representative of low-shear conditions for complex fluids. Three independent measurements were performed for each composition.

The water absorption test (Cobb) of kraft paper was carried out with adaptations of the TAPPI T441 om-20 standard [28]. To carry out the analysis, a nanoreinforced sodium silicate adhesive was used instead of water, as recommended by the aforementioned standard. One hundred milliliters of sodium silicate adhesive reinforced with 0.5% (wt.%) NA, 0.5% (wt.%) NS, and 0.5% (wt.%) kaolin (each of them separately) was poured into a metal ring apparatus with a diameter of 100 mm containing a kraft paper sample with dimensions of 150 × 150 mm fixed to the lower end of the ring. After being poured, the nanoreinforced adhesive remained in contact with the kraft paper for a period of 120 s, after which the nanoreinforced adhesive contained inside the ring was removed. The paper sample was weighed before and after the process. The Cobb test is essential for understanding barrier behavior through the relationship between the wetted area (g/m^2^) and the mass of the nanoreinforced adhesive that is absorbed by the paper. To carry out the analysis, five repetitions were performed for each composition evaluated.

The wettability of the paper surface was analyzed by determining the contact angle using the sessile drop technique (Krüss DSA25, Hamburg, Germany) with DSA3 1.0 software. Drops (~10 μL) of both unreinforced and nanoreinforced sodium silicate adhesives (containing NA, NS, and kaolin) were deposited, and measurements were acquired every second over a period of 60 s. The initial contact angle was measured at 5 s, while the final value was obtained at 60 s. Twenty-five replicates were conducted per composition, resulting in 150 total measurements.

### 2.4. Composite Production

The production of composites was carried out following the methodology developed by Faria et al. [6]. The composites were manufactured by bonding 20 layers of recycled kraft paper using a nanoreinforced sodium silicate adhesive, applied along the glue line in solution form, with an application rate of 132.65 g/m^2^.

**Figure 2:**
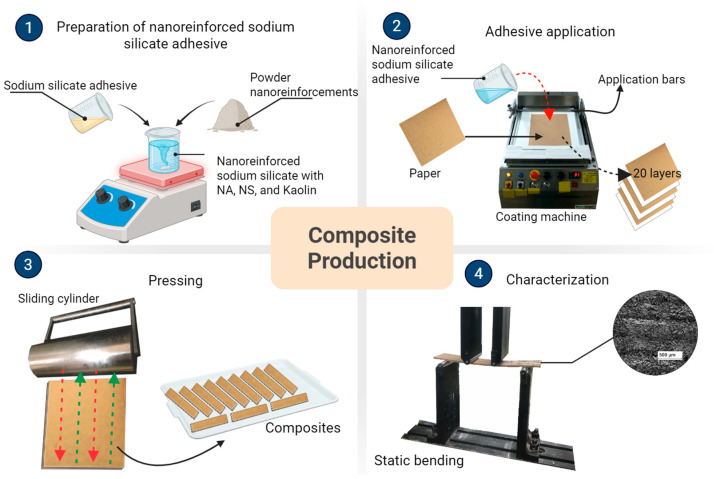
Composite production process.

Uniform adhesive application was achieved using a Mathis SVA-IR paper coating machine equipped with a 100 µm bar and balanced loads of 100 g on each side to ensure stability and consistent spreading at a speed of 4 m/min. The process was conducted under controlled ambient conditions (19 ± 2 °C). After coating, the paper layers were pressed using a stainless steel cylindrical roller (10.8 kg, 89 mm diameter, 205 mm width), corresponding to a contact area of 500 mm^2^ and an applied pressure of 0.215 MPa. The overall composite manufacturing procedure is presented in Figure 2.

Compaction was achieved using a sliding cylinder operating at a constant speed of 4 m/min under reciprocating motion (0.43 MPa), applying a pressure of 4.30 MPa to each composite. The pressing procedure was conducted after every two glue lines. Based on earlier work [6], these parameters ensured optimal mechanical performance. The composites were manufactured with and without nanomaterial incorporation as reinforcement in the sodium silicate matrix. A 0.5% nanoreinforcement content in the adhesive was established on the basis of previous tests and corroborated by the results obtained by Furtini et al. [27], who reported high agglomeration of the reinforcement phase for contents greater than 1%.

For each composition studied, 2 composites were produced, and the experimental design is presented in Table 2.

Once the paper layers were bonded, the assembly mass was recorded using a precision balance with a resolution of 0.0001 g. The samples were then compressed between perforated metal plates using clips to ensure a final thickness of approximately 6 mm and conditioned for 24 h at room temperature. The composites were subsequently oven-treated at 85 °C for 48 h to finalize sodium silicate polymerization. Dry mass and dimensions were measured using a digital caliper to allow calculation of the basis mass per adhesive layer. The paper grammage (*Gp*) was initially determined according to Equation (1).

*Gp* (g/m^2^) = *msp*/*Ap* (1)

where *msp* corresponds to the oven-dried mass of the paper at 100 °C for 30 min (g), and *Ap* is the paper area (m^2^).

The total adhesive amount in the composites after pressing (*Gta*) was determined using Equation (2).

*Gta* (g/m^2^) = (*msc*/*Ac*) − *Gp* × 20 (2)

where *msc* is the dry mass of the composite after drying in an oven at 85 °C for 48 h (g), *Ac* is the area of the composite (m^2^), and 20 is the number of paper layers in the composite.

The adhesive mass per glue line present in the composites after pressing (*Glc*) was determined using Equation (3).

*Glc* (g/m^2^) = *Gta*/*ngl* (3)

where *Gta* is the total adhesive grammage (g/m^2^) and *ngl* is the number of glue lines equal to 19.

Following fabrication, the composites were maintained under controlled environmental conditions (20 ± 3 °C and 65 ± 5% relative humidity) until the adhesive curing process was complete. The samples were then machined into two specimens with dimensions of 180 mm × 40 mm × 6 mm.

### 2.5. Characterization of the Composites

#### 2.5.1. Physical Characterization

The apparent density of the composites was evaluated using four specimens measuring 180 mm × 40 mm × 6 mm. Equation (4) was used to obtain the apparent density.

Da (g/cm^3^) = *m*/*v* (4)

where *m* is the mass of the sample (g) and *v* is the volume of the sample (cm^3^).

#### 2.5.2. Mechanical Characterization

Four samples of each composition evaluated with dimensions of 180 mm × 40 mm × 6 mm were used to obtain the modulus of rupture (MOR), modulus of elasticity (MOE), limit of proportionality (LOP), and toughness (TE) properties by four-point static bending tests, with the dimensions of the samples and the test parameters used on the basis of the recommendations of RILEM [29]. The samples were tested in an Arotec universal servoelectric testing machine equipped with a 20 kN load cell, with a testing speed of 10 mm/min.

The mechanical properties were obtained using Equations (5)–(7) as described by Tonoli et al. [30] and Equation (8) as described by Garcia [31].

MOR (MPa) = (*P_max_* × *L*)/(*b* × *h*^2^) (5)

LOP (MPa) = (*P_lop_* × *L*)/(*b* × *h*^2^) (6)

MOE (MPa) = ((276 × *L*^3^)/(1296 × *b* × *h*^3^)) × *m* (7)

TE (N.mm/mm^3^) = *Energy*/(*b* × *h*) (8)

where *P_max_* represents the maximum applied load (kN); *L* is the span length (135 mm); *b* and *h* denote the width and thickness of the specimen (mm), respectively; *P_lop_* corresponds to the load at the upper point of the linear segment of the load–deflection curve (kN); and *m* is the slope of the straight line associated with the elastic behavior of the material. *Energy* corresponds to the energy absorbed per cross-sectional area during the bending test.

#### 2.5.3. Scanning Electron Microscopy (SEM)

The fracture surface resulting from the bending test was analyzed by field emission scanning electron microscopy (SEM-FEG). The specimens were gold-coated in an evaporator before observation using a Tescan Clara SEM (Brno, Czech Republic) operating at 20 kV.

### 2.6. Statistical Analysis

A completely randomized design was employed for the statistical evaluation of the multilayer paper composites. The dataset was analyzed by ANOVA, followed by Tukey’s test at the 5% significance level, to identify the effects of nanomaterials on the physical and mechanical properties. All statistical analyses were conducted using Sisvar 5.6 software.

## 3. Results and Discussion

### 3.1. Characterization of the Kraft Paper and Nanomaterials

#### 3.1.1. FTIR

**Figure 3:**
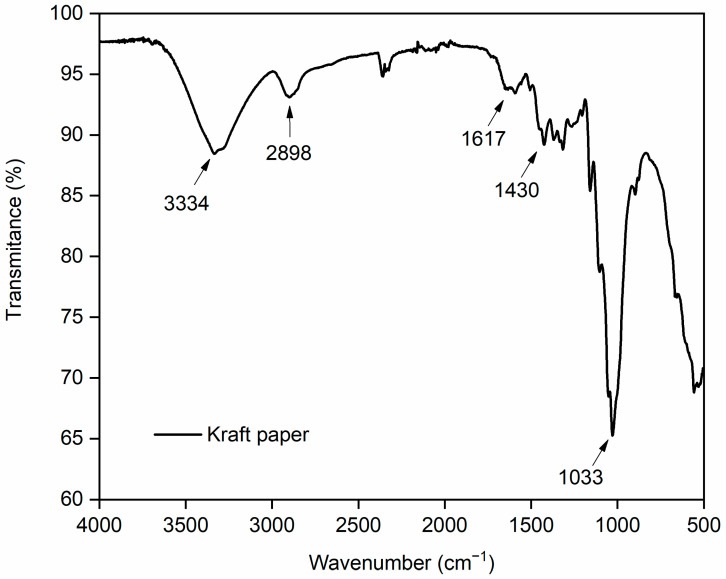
FTIR spectra of the kraft paper.

The FTIR test is highly relevant for verifying the molecular structures presented in the paper, directly relating it to its physical and mechanical properties [32]. The FTIR spectra for the kraft paper are shown in Figure 3.

Based on the FTIR spectrum of the kraft paper used in composite production, it is possible to observe important characteristic peaks present in the spectrum. The intense peak observed at 3334 cm^−1^ is characteristic of N–H stretching vibrations of carbohydrates, adsorbed water, and proteins, as well as O–H functional groups present in cellulose [33,34,35]. The O-H chemical groups present in the structure of kraft paper bind to the siloxo functional groups (Si-O) present in the sodium silicate adhesive during the production of the composites, subsequently forming silanol bonds (Si-OH) [36].

Additionally, in the FTIR spectrum, there are other characteristic peaks of cellulose, such as the peak at 2898 cm^−1^, attributed to the asymmetric stretching vibrations of the hydrophobic CH_2_ [37]. Consistent with literature reports, this peak is attributed to C–H absorption related to polymers in plant fibers and extractive components. Extractives are low-molecular-weight compounds (in contrast to wood macromolecules), which include a wide range of compounds, most of which are secondary metabolites (i.e., compounds that are not essential for plant growth). Although present at low concentrations in softwoods (2–5%) and hardwoods (3–8%), these substances may influence the adhesive–adherend interfacial interaction by altering surface polarity, wettability, and permeability, as well as by modifying the curing and setting behavior of the adhesive system [38]. Furthermore, the type of extractives present in lignocellulosic materials plays a critical role in the adhesion process. Acidic or alkaline extractives may alter the curing behavior of wood adhesives, either accelerating or retarding their setting [39].

Furthermore, the peak observed at 1617 cm^−1^ is associated with the symmetric in-plane C=C aromatic stretching vibration characteristic of lignin, which is responsible for increasing the rigidity and resistance to degradation of the plant fiber [40]. The peak at 1430 cm^−1^ is often associated with CH_2_ bending vibrations of cellulose [41,42]. The peak at 1033 cm^−1^ is attributed mainly to the C–O stretching of carbohydrates present in plant materials [43].

**Figure 4:**
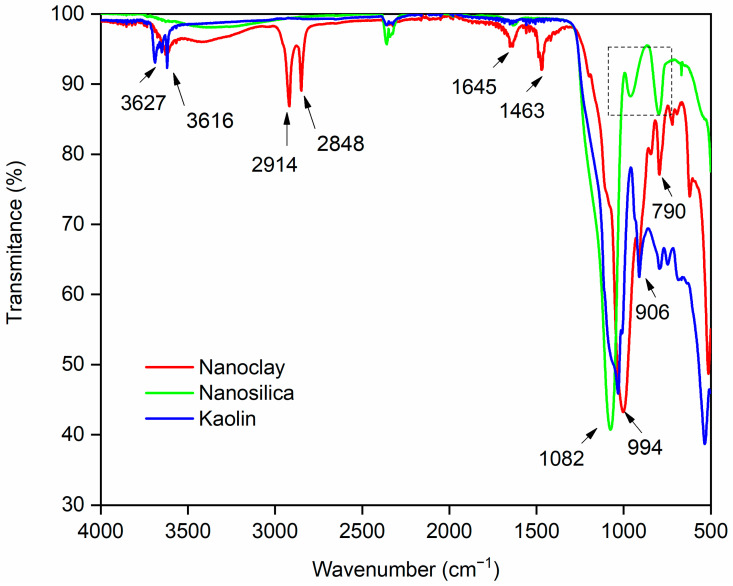
FTIR spectra of the nanoclay, nanosilica, and kaolin.

Regarding the nanomaterials, Figure 4 presents the FTIR spectra for nanoclay, nanosilica, and kaolin.

On the basis of the FTIR spectrum, characteristic peaks for the nanomaterials under study can be seen. For nanoclay, the peak at 3627 cm^−1^ is attributed to the OH- stretching vibration; at 2914 and 2848 cm^−1^, there are typical peaks of methylene symmetric vibrations; C=O in 1645 cm^−1^ and C-O-H in 1463 cm^−1^, which already corresponds to the stretching vibration of the Si–O bond at 994 cm^−1^; the peak at 790 cm^−1^ may correspond to the stretching vibration of Al–O–Si, and the peak at 531 cm^−1^ is due to a Si–O bending vibration [44,45,46]. In terms of nanosilica, there is an intense peak at 1082 cm^−1^ related to Si-O-Si asymmetry; at 955 and 801 cm^−1^ (identified by the dashed rectangle in Figure 4), Si-O-Si symmetry peaks may be related to the stretching and bending vibrations of Si-O-Si at 503 cm^−1^, which are characteristic of silica [47,48]. For kaolin, there are characteristic peaks at 3627 and 3616 cm^−1^ corresponding to the stretching frequencies of the OH groups of water molecules because of the presence of water in kaolin [44,49]; at 1027 cm^−1^, an intense peak corresponding to the stretching of Si-O or Al-O is observed, whereas at 906 cm^−1^, a peak related to the bending of the Al-OH bond is observed, and at 790 cm^−1^, the peak verified is attributed to the stretching of the Si-O-Si bond, representing kaolin quartz [49]. By comparing the three nanoreinforcements (Figure 4), it is clear that the nanoclay presented peaks related to the symmetrical vibration of the methylene functional group (2914 and 2848 cm^−1^) that did not occur for nanosilica and kaolin. This behavior is attributed to the enhanced ability of nanoclay to interact with organic groups, owing to its lamellar structure, which is characterized by a high specific surface area, elevated cation exchange capacity, and the presence of permanent negative charges arising from isomorphic substitutions [50].

#### 3.1.2. SEM

**Figure 5:**
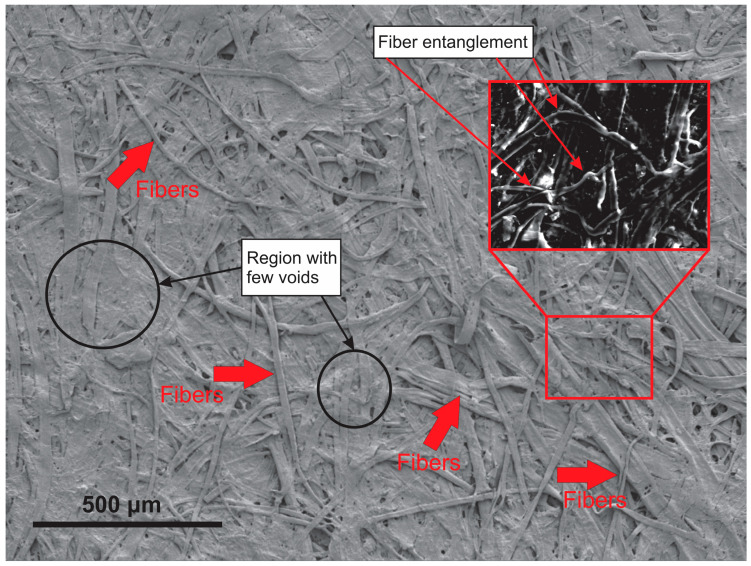
Electron micrograph obtained by SEM of the surface of recycled kraft paper.

The fibers that make up the paper samples analyzed by SEM were random and appeared to be consistent with those of kraft paper (Figure 5), as verified by several authors [51,52,53].

The morphology presented in Figure 5 highlights important considerations regarding the surface of the kraft paper used in the production of the composites. As seen, the presence of zones containing voids located at the intersection of the fibers favors the penetration of the adhesive and, consequently, a better interaction with the paper layers through physical and chemical bonds, in addition to mechanical anchoring. Unlike a surface with low roughness, the electron micrograph of kraft paper was favorable for efficient covering of the paper by the adhesive. Surface roughness significantly influences the adhesion of cellulosic materials, exhibiting positive or negative effects depending on the application and type of adhesive used. Rougher surfaces can increase the effective contact area, promote mechanical interlocking, and hinder crack propagation, resulting in stronger bonds. On the other hand, excessive levels of roughness may require greater adhesive penetration into microcracks, which, if not adequately achieved, can compromise both bond strength and protection against environmental agents. This behavior was confirmed by Afferrante et al. [54], who reported that successive increases in surface roughness tended to reduce mechanical strength values during pull-off testing. Thus, the surface roughness is optimal for the maximum mechanical strength of multilayer paper composites bonded with adhesive, which depends on the substrate properties and the physicochemical characteristics of the adhesive, such as the pH, viscosity and solid content [55].

**Figure 6:**
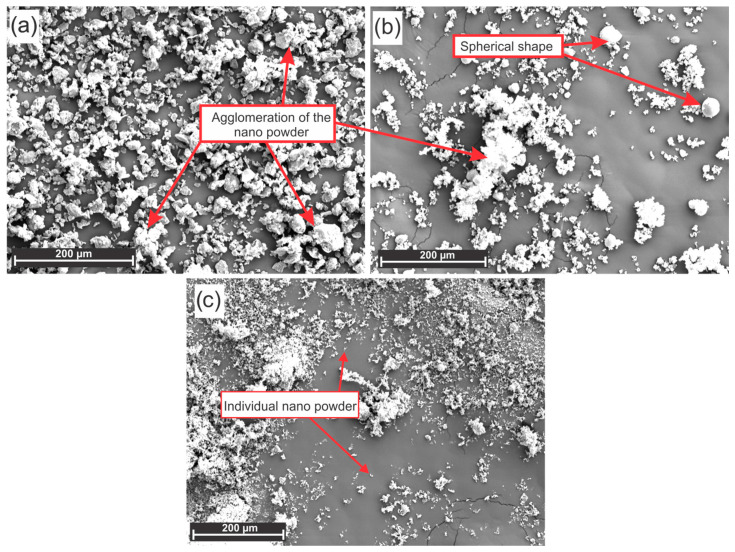
Electron micrographs obtained by SEM of the nanomaterials: (**a**) NA, (**b**) NS, and (**c**) kaolin.

In addition to the morphology of the substrate (kraft paper), the topographic characteristics of the nanomaterials under study strongly influence the properties of the composites, especially the mixture of the nanoreinforcements with the sodium silicate adhesive (Figure 6).

The topographic profiles of the nanomaterials under study revealed that the clay nanoparticles and silica nanoparticles were almost completely agglomerated, whereas the kaolin was unique to the powdered material, albeit with some agglomerated parts. The clay nanoparticles have an irregular shape with edges, whereas the silica and kaolin nanoparticles have a spherical shape. A more spherical surface of the nanomaterials is desired to achieve better distribution and interaction with sodium silicate, as the closer the nanoreinforcement is to a sphere, the greater its contact area. The contact area between the reinforcement and the matrix in a composite material is crucial for load transfer and, consequently, for the mechanical properties of the final material [56]. A good contact area and strong adhesion between the reinforcement and the matrix are essential for the matrix to transfer stress to the reinforcement, thereby increasing the composite strength and stiffness.

### 3.2. Characterization of the Sodium Silicate Adhesive Reinforced with Nanomaterials

#### 3.2.1. Thermal Properties

**Figure 7:**
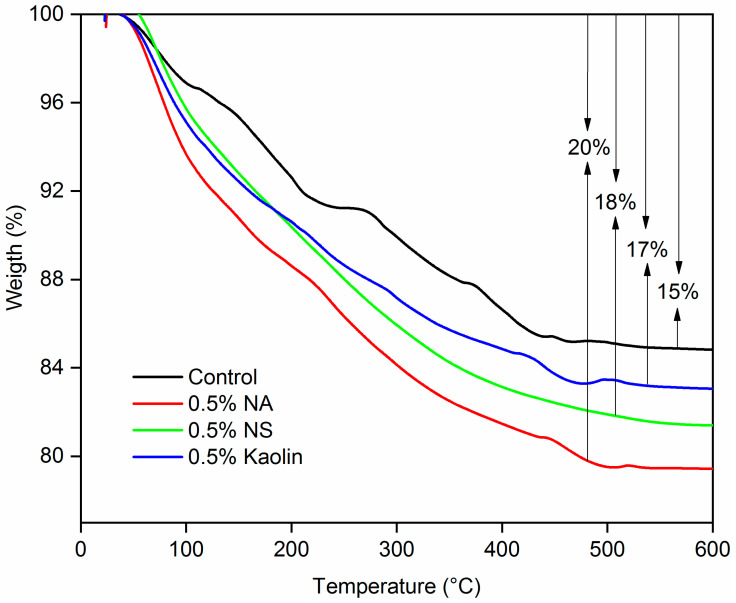
TGA curves of pure sodium silicate reinforced with NA, NS, and kaolin.

The thermal stability of sodium silicate nanoreinforced with 0.5% NA, 0.5% NS, and 0.5% kaolin obtained by TGA thermogravimetric analysis is shown in Figure 7. The initial mass decline is primarily attributed to water evaporation from the sodium silicate, occurring between 40 and 140 °C [57,58]. A similar pattern was identified by Orji et al. [35], with water loss spanning from 40 to 170 °C.

The thermal degradation curves of the nanoreinforced sodium silicate subjected to a thermal sweep between 25 and 600 °C are shown in Figure 7. Using the thermogravimetric curves, it was possible to verify that the total mass loss for pure sodium silicate was 15%; for sodium silicate reinforced with 0.5% NA, it was 20%, whereas for sodium silicate containing 0.5% NS, it was 18%; for sodium silicate with 0.5% kaolin in the composition, the total mass loss was 17%. The thermal degradation curves of the nanoreinforced sodium silicate showed a loss of mass at approximately 60 °C; that is, after this point, the adhesive denatured. Within the temperature range of approximately 150–300 °C, the removal of more strongly bound water and species associated with silanol (Si–OH) groups subsequently occurs, involving progressive dehydration processes and the onset of condensation within the silicate network [59]. In the intermediate range of 300–500 °C, the TGA curves for all the compositions exhibit lower mass loss, which is associated with the removal of residual structural water and the intensification of polycondensation reactions, leading to the formation of a more stable three-dimensional Si–O–Si network. Furthermore, thermal stability is observed from 480 °C until the end of the thermal scan, demonstrating that the sodium silicate became solid and possibly transformed into solid metasilicate [60].

As evidenced by the TGA curves in Figure 7, the incorporation of nanomaterials into sodium silicate adversely affected the thermal stability of the adhesive. The char residue increased from 15% for the pure adhesive to 20% for the nanoclay-modified system. This behavior can be explained by the nature of nanoclay, which exhibits a residual mass of approximately 70%, as reported by Rao et al. [61], corresponding to a mass loss of 30%. Comparable effects were also noted for nanosilica and kaolin.

#### 3.2.2. Rheological Properties

**Figure 8:**
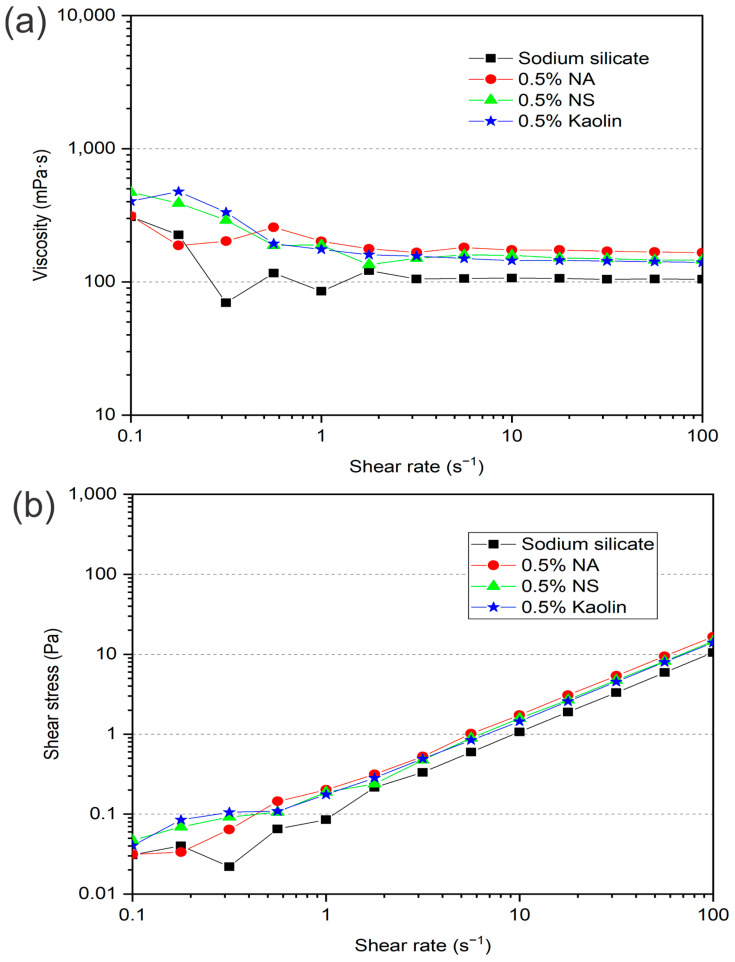
Viscosity (**a**) and shear stress (**b**) profiles as a function of shear rate for the various compositions analyzed.

The results of the analysis of the rheological properties of the sodium silicate adhesive reinforced with NA, NS and kaolin are shown in Figure 8.

According to Figure 8a, at shear levels lower than one, all the adhesives exhibited viscosity variation. For example, for the pure sodium silicate adhesive, instability of viscosity values is observed, with the viscosity varying from 308 mPa·s in 0.1 s^−1^ to 69 mPa·s in 0.3 s^−1^; however, at 0.5 s^−1^, the fluid behavior is stable. The same behavior was observed for the other adhesives reinforced with nanomaterials, all of which exhibited viscosity stability under shear rates greater than 0.5 s^−1^. From the viscosity curve behavior shown in Figure 8a, these adhesives can be classified as Bingham plastic fluids since they exhibit a reduction in viscosity at low shear rates, followed by stabilization of values as the shear rate increases [62].

The classification of pure sodium silicate and nanoreinforced adhesives as Bingham plastic fluids is further supported by the shear stress–shear rate curves, as shown in Figure 8b. Fluids defined as Bingham plastics are a type of non-Newtonian fluid that behaves as a solid under low stress but flows as a liquid under high stress. They require a minimum of stress, known as yield stress, to initiate flow. Despite being a linear representation, the Bingham plastic model does not adequately depict the rheological behavior of the Bingham plastic fluid in the low shear rate range [63,64]. Sodium silicate-based adhesives, which are aqueous solutions composed predominantly of SiO_2_ and Na_2_O, along with various organic and inorganic particulate additives, exhibit intense particle interactions that influence their rheological behavior [6]. Therefore, on the basis of the obtained rheological results, the addition of nanomaterials did not cause significant changes in the viscosity of the nanoreinforced adhesives or maintain the mobility properties necessary for flow, transfer, penetration, and substrate wetting, thereby ensuring the preservation of the mechanical performance of the composites [65].

To ensure a sufficiently strong bond, it is essential that the adhesive adequately penetrates the wood surface, promoting intimate molecular contact with the substrate [66]. Preserving the rheological properties of sodium silicate-based adhesives is of fundamental importance for their practical application, as these characteristics directly influence their performance during processing and adhesion to the substrate. A proper balance of adhesive viscosity is crucial for optimizing the manufacturing process of multilayer paper composites, ensuring a homogeneous distribution of the adhesive and appropriate penetration into the substrate surface. Reducing the viscosity of the nanoreinforced adhesive may result in excessive penetration of the adhesive, compromising the formation of the adhesive interface between the paper layers. On the other hand, high viscosity hinders the spread and promotes a nonuniform distribution of the adhesive on the paper surface, impairing the bond strength and, consequently, the mechanical performance of the composites [67].

#### 3.2.3. Adhesive Absorption Properties

**Figure 9:**
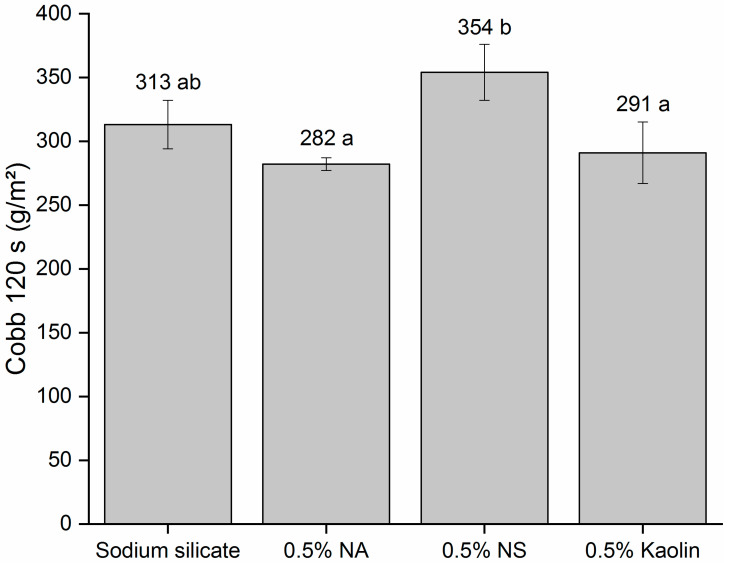
Absorption of pure and nanoreinforced sodium silicate adhesive by kraft paper (adapted Cobb test). Averages followed by the same letter indicate no significant difference according to the Tukey test (*p* > 0.05).

In addition to the viscosity of the adhesive, another relevant factor in its practical use occurs through the absorption of the adhesive by the substrate, in this case, by kraft paper. The results of the absorption of pure adhesive and that reinforced with nanomaterials by paper according to the adapted Cobb test are shown in Figure 9.

The Cobb test is traditionally used with water to measure the amount of this substance absorbed at a specific time per 1 m^2^ of paper under 1 cm of water [28]. In the adaptation carried out in the present work, water was replaced by pure and nanoreinforced sodium silicate adhesive to understand the absorption behavior of the adhesive. On the basis of the results reported for the adapted Cobb test, significant differences were observed between the adhesives evaluated. The sodium silicate adhesive containing 0.5% NS differed statistically from the compositions containing 0.5% NA and 0.5% kaolin but was statistically equal to pure sodium silicate. This higher Cobb value observed over a period of time of 120 s indicates that the composition containing 0.5% NS showed greater adhesion absorption by kraft paper [68]. However, notably, the absorption of the nanoreinforced adhesive with NS was statistically equal to that of pure sodium silicate in the Cobb test; that is, for practical purposes, the quality of the glue line in terms of the amount of adhesive present was not influenced.

#### 3.2.4. Wettability Properties

**Figure 10:**
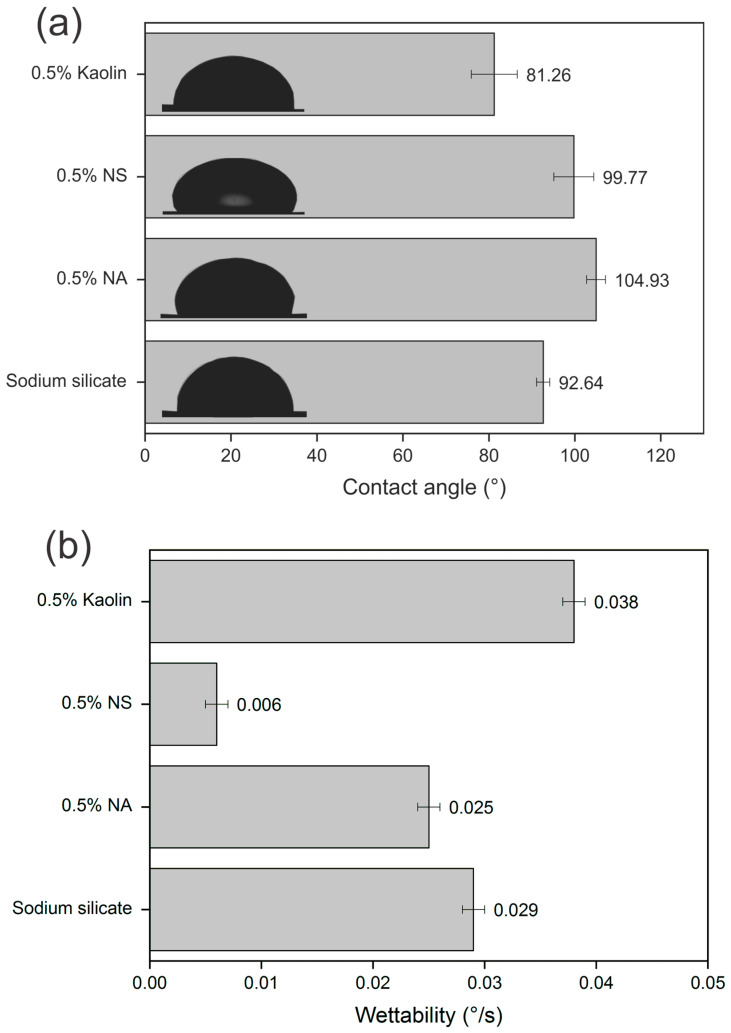
(**a**) Contact angle and (**b**) wettability for pure and the nanoreinforced sodium silicate adhesive. The drop images are on the same scale.

The mechanism of adhesive spreading over the kraft paper surface can be further elucidated through contact angle and wettability analyses performed using the sessile drop method (Figure 10). The nanoreinforced adhesive with 0.5% kaolin presented a contact angle below 90°, indicating hydrophilic behavior at the interface [69].

The insertion of kaolin into the sodium silicate adhesive reduced the contact angle, a behavior justified by the chemical composition of the kaolin under study, composed mainly of kaolinite (Al_2_O_3_·2SiO_2_·2H_2_O), which has two water molecules in its composition, causing the water present in the adhesive containing kaolin to establish interactions with the hydroxyl groups of kraft paper. With respect to the contact angle for the other compositions, all of them resulted in a hydrophobic character of the nanoreinforced adhesive in contact with the paper, since the angle formed in the contact of the liquid with the solid surface was greater than 90° [70]. Therefore, reducing the contact angle is directly associated with increasing the wettability of the adhesive on the paper surface, which, in turn, promotes the formation of more adhesive and efficient interfaces [71].

The incorporation of 0.5% nanosilica (NS) into the sodium silicate-based adhesive resulted in a slight variation in the contact angle compared with that of the unmodified adhesive; however, there was a significant change in the wettability of the nanoreinforced adhesive, indicating relevant changes in its interaction with the substrate surface. Wettability corresponds to the ability of the adhesive droplet to spread over the substrate between the first 5 s of contact with kraft paper and the subsequent 55 s, reflecting the dynamic spreading behavior of the adhesive over time [6]. The kaolin-reinforced nanoadhesive spread more strongly (°/s) on the paper surface, which can be attributed to the intrinsic characteristics of this nanomaterial used as a reinforcement for sodium silicate. On the other hand, the NS-reinforced nanoadhesive exhibited the lowest degree of spread (°/s) during the wettability test. This behavior can be attributed to the agglomeration of NS particles within the sodium silicate adhesive, which persisted despite constant agitation, preventing these particles from penetrating the pores of the paper upon contact with the adhesive system. This behavior can be explained by the rheometric curves of the nanoreinforced adhesive. As shown in Figure 8a, large viscosity values are observed at low shear rates. This behavior was reported by Furtini et al. [27], who reported an increase in viscosity from 22 to 294 Pa·s for a pure cardanol-formaldehyde adhesive and a nanoreinforced adhesive with 1% NS, respectively. Owing to the hydrophilic nature of silica nanoparticles, they tend to agglomerate easily because of the large number of hydroxyl groups that react with the water present in the adhesive [72].

Regarding the sodium silicate adhesive modified with 0.5 wt.% NA nanoparticles, a hydrophobic behavior similar to that of the pure sodium silicate adhesive was observed. The incorporation of nanoparticles as reinforcements in adhesives for bonding paper and wood layers to produce composites with structural functions is an innovative strategy to improve the properties of these materials for their use in various technological applications. Pelit et al. [8] evaluated the efficiency of laminated composites produced with 4 mm thick layers of poplar and beech wood bonded with polyvinyl acetate (PVAc), urea-formaldehyde (UF), and epoxy adhesives. The adhesives were enriched with varying concentrations of different nanoparticles, including graphene (0.25% and 0.50%), silica (1% and 2%), and cellulose (1% and 2%). According to the authors, the addition of all the nanoparticles improved the performance compared with that of the base adhesives. The addition of graphene significantly improved the MOE, MOR, and compressive strength (CS), whereas the addition of cellulose and silica primarily increased the flexural strength (BS).

### 3.3. Characterization of the Composites

#### 3.3.1. Physical Properties of the Composites

Paper grammage is a critical factor governing the performance of composite materials. The kraft paper used herein presented a grammage of 182 ± 6 g/m^2^. The total adhesive grammage and the grammage per glue line for each formulation are listed in Table 3. The total adhesive grammage refers to the aggregate adhesive applied across the 19 glue lines.

The production of composites with layers of recycled kraft paper bonded with pure sodium silicate adhesive (Control), as well as composites produced with nanoreinforced adhesive with NA, NS and kaolin, did not differ in terms of the total amount of adhesive in the entire composite or per glue line. Maintaining this stability is essential, since mass homogeneity among the composites ensures uniform glue line thickness, reflecting their physical and mechanical characteristics.

Adhesive viscosity is directly correlated with the basis weight per glue line, as increased viscosity impairs uniform spreading on the paper surface, leading to higher adhesive deposition per bond line. As verified in Figure 8a, all the evaluated formulations of the sodium silicate adhesive reinforced with inorganic materials showed similar viscosity behaviors, justifying the close adhesive basis weight values in the glue line of the composites. Notably, the final basis weight observed in the composites was much lower than the initial basis weight of the applied nanoreinforced sodium silicate adhesive solution (132.65 g/m^2^).

The difference in adhesion values before and after the production of the composites can be explained by two main factors: (1) the sodium silicate adhesive solution contained ~49% solids, so during the adhesive polymerization stage, the water evaporated, resulting only in polymerized adhesion at the glue line; (2) in the production of the composites, the kraft paper layers bonded with the nanoreinforced adhesive were pressed at 0.215 MPa, causing some of the adhesive present at the glue line to be expelled from it. The number of pressing cycles is among the critical parameters for ensuring the quality of the final product and influences the physical and mechanical properties of the composite. Its influence extends from compaction of the paper layers to adequate adhesion of the adhesive, directly impacting the final properties of the material [10,73].

**Figure 11:**
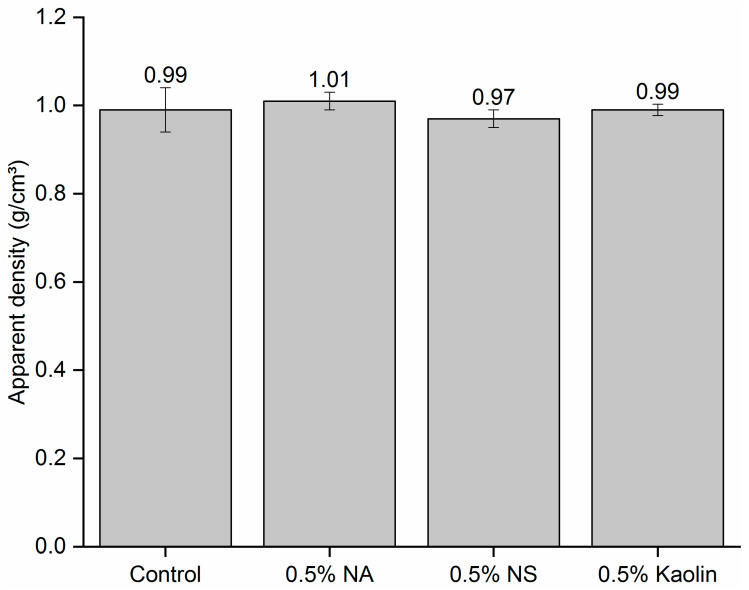
Apparent density of the composites produced. Averages followed by the same letter indicate no significant difference according to the Tukey test (*p* > 0.05). ns indicates a nonsignificant *p* value at the 5% level.

The apparent density of the produced composites is shown in Figure 11. The mechanical properties are directly correlated with the apparent density of the composites, as well as with the strength of the adhesive bond line formed between the sodium silicate nanoreinforced with NA, NS, and kaolin and the paper layers.

As shown in Figure 11, no significant differences were found between the compositions evaluated. This outcome highlights the uniform nature of the composites, irrespective of the nanomaterial used. The consistent apparent density among all compositions can be primarily attributed to the low reinforcement content (0.5%). Although the density of sodium silicate is between 1.368 and 1.381 g/cm^3^ and that of nanosilica is 0.083 g/cm^3^, the insertion of 0.5% nanosilica, even though it occupies a larger volume in the composite, has little effect on the density.

This same behavior was verified by Dinesh et al. [26] in epoxy matrix composites reinforced with kenaf and banyan fibers in addition to nanosilica insertion content. The authors reported that the insertion of 0.5% (wt.%) NS into the composites did not affect their density; however, for contents of 1% and 2% (wt.%), the density decreased as the content of NS inserted into the composites increased. Moreover, compared with that of the neat epoxy matrix, the porosity of the composites increased, a behavior directly associated with the presence of voids generated by particle agglomeration within the composites. As shown in Figure 6, NA particles exhibited a pronounced tendency toward agglomeration, corroborating the observations reported by the authors.

#### 3.3.2. Mechanical Properties of the Composites

**Figure 12:**
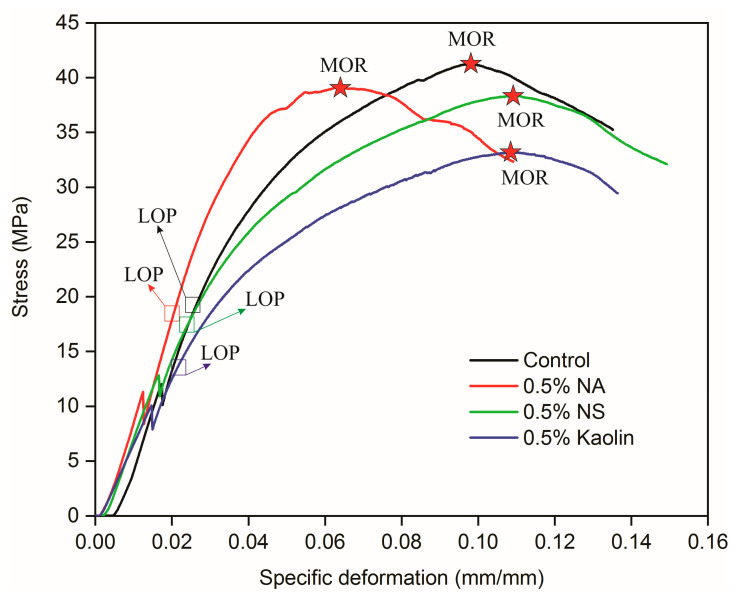
The characteristic stress–strain behavior of the composites under static bending is displayed, with red stars identifying the MOR values.

The mechanical properties of static flexural strength, stiffness, and toughness are important in the selection of materials for technological applications, influencing performance under operational loads and the reliability of the developed product [74]. Figure 12 presents the representative stress–strain behavior obtained from static bending tests of the analyzed composites.

From the stress–strain curves, key mechanical properties of the composites can be determined, including stiffness, strength, and energy absorption capacity up to fracture. As observed from Figure 12, the LOP were very similar; however, the behavior from the elastic to plastic phase during mechanical stress in the composites underwent a slight change. Similarly, the points related to the MOR of the composites were similar, with the exception of the composites containing kaolin. On the basis of the results from the stress × strain curves, it was possible to extract the results of the mechanical properties obtained from the static bending test (Table 4).

As shown in Table 4, no statistically significant differences were observed among the evaluated formulations for MOR and LOP. However, the MOE and toughness significantly varied. These results are consistent with those reported by Liu et al. [17]. The authors reported a 74% increase in bending strength for laminated wood composites bonded with a sodium silicate adhesive nanoreinforced with inorganic materials compared with unmodified formulations. The stability observed in the MOR and LOP results can be primarily attributed to the homogeneity achieved in the apparent density of the composites (Figure 11), which, in turn, resulted from the consistent adhesive grammage across the bonding lines.

A significant impact on composite stiffness was observed with the incorporation of NSs and kaolin. While the MOE values were similar between the composites produced with sodium silicate reinforced with NA and those produced without NA, those produced with NS and kaolin decreased by 12 and 23%, respectively. This finding implies that composites fabricated with 0.5% NA and those without nanoreinforcement present higher resistance to elastic deformation, since elevated MOE values indicate increased rigidity and diminished deformation under stress [52,75].

In addition to the significant differences observed among the formulations in terms of composite stiffness, a pronounced increase in toughness was also evident for the composites containing 0.5% NA, NS, and kaolin. Compared with those produced with the control formulation, the toughness of the composites produced with the sodium silicate adhesive reinforced with 0.5% NA and NS significantly increased by 25%. This increase in toughness is directly related to the ability of composites to absorb energy and resist the formation and propagation of cracks, significantly reducing the occurrence of catastrophic failure [76]. This increase in toughness is highly important in laminated composites, since it provides greater resistance to delamination, increasing the load support and mechanical stability of the composite over time [77].

**Figure 13:**
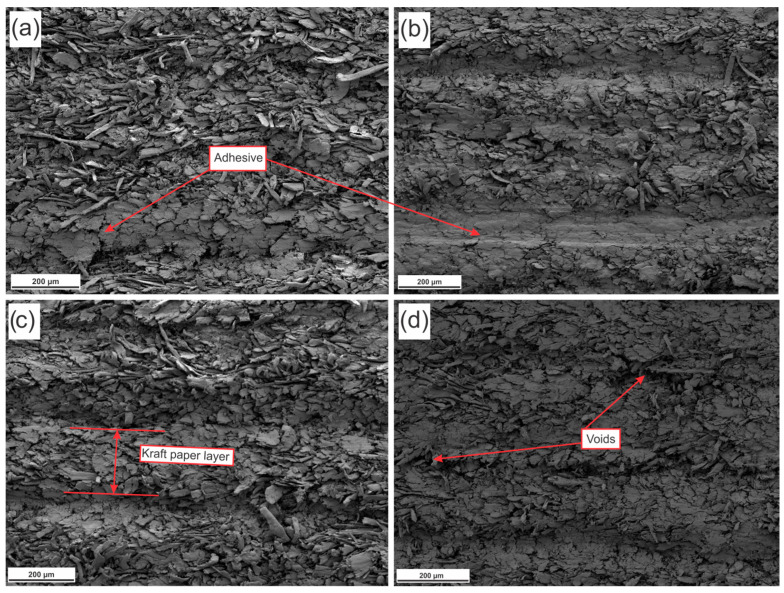
Electron micrographs of the fracture surface from the cross section of the composites obtained by SEM. (**a**) Control; (**b**) 0.5% NA; (**c**) 0.5% NS; (**d**) 0.5% kaolin.

The adequate mechanical behavior can be mainly ascribed to the existence of a homogeneous and continuous glue line along the entire bonding interface, directly linked to the adhesive grammage per glue line. Figure 13 displays SEM images of the composite cross-sections following the static bending test.

SEM analysis demonstrated that the fracture surfaces were uniform across the paper layers constituting the composites. The presence of an intact and homogeneous glue line indicates efficient stress transfer between the paper layers and the adhesive under mechanical loading. Accordingly, the electron micrographs revealed no evidence of interlayer delamination during the static bending test for the evaluated formulations, confirming adequate interfacial adhesion between the bonded substrates. The presence of voids between the layers of kraft paper that make up the composites produced with 0.5% kaolin is shown in Figure 13d. This porosity may have contributed to the reduction in MOE values, along with the greater wettability of this formulation (Figure 10b).

The mechanical properties obtained in the present study are consistent with those reported by Faria et al. [6], who evaluated the physical, mechanical, and microstructural performance of multilayer paper composites manufactured from kraft paper bonded with sodium silicate adhesive nanoreinforced with cellulose nanofibrils (CNFs). The authors reported MOR values ranging from 38.8 MPa for the formulation without reinforcement to 46.7 MPa for composites containing 0.5% CNFs. The strength increase was attributed to the formation of a percolated CNF network within the sodium silicate matrix, stabilized by hydrogen bonding interactions.

## 4. Conclusions

The results of the present study revealed that the insertion of 0.5% NA and 0.5% NS led to a significant 25% increase in the toughness of the composites compared to those produced with unreinforced sodium silicate adhesive. This improvement was achieved owing to a deep understanding of the behavior of the nanoreinforced adhesive using different characterization techniques, such as FTIR, which identified the main functional groups and their respective intensities, as well as rheological, barrier, and absorption analyses of the nanoreinforced adhesive by kraft paper, which provided valuable information about the process of spreading and absorption of the adhesive in contact with the paper.

The integration of nanomaterials into sodium silicate adhesives resulted in superior mechanical performance of the composites, thereby extending the application potential of multilayer paper products and promoting sustainable resource utilization. As a result, nanoreinforced multilayer paper composites can be considered a viable alternative to conventional materials.

## Figures and Tables

**Table 1:** Physicochemical parameters of the sodium silicate adhesive. * Data provided by the adhesive producer.

Parameter	Sodium Silicate *
Na_2_O concentration (%)	8.4–8.6
SiO_2_ concentration (%)	27.5–27.9
Total concentration (%)	36–37
Silica module (SiO_2_:Na_2_O)	3.25–3.30
Specific density (g/cm^3^)	1.368–1.381
Viscosity (cP)	70–170
Turbidity (NTU)	0–5 NTU
Iron content (ppm)	<60

**Table 2:** Experimental design of the composites produced.

Compositions	Description
Control	Control composite
0.5% NA	Composite with adhesive reinforced with 0.5% montmorillonite nanoclay
0.5% NS	Composite with adhesive reinforced with 0.5% nanosilica
0.5% Kaolin	Composite with adhesive reinforced with 0.5% kaolin

**Table 3:** Total adhesive grammage and adhesive grammage per line of glue.

Composition	Total Adhesive Grammage (g/m^2^)	Grammage per Glue Line (g/m^2^)
Control	1249 ± 72 *	66 ± 4
0.5% NA	1291 ± 89	68 ± 5
0.5% NS	1228 ± 42	65 ± 2
0.5% Kaolin	1247 ± 25	66 ± 1

* Standard deviation.

**Table 4:** Mechanical properties of the composites in the static bending test.

Composition	MOR (MPa)	LOP (MPa)	MOE (MPa)	Toughness (N.mm/mm^3^)
Control	38.1 ± 4.4 * a	19 ± 1.8 a	5272 ± 552 ab	0.0012 ± 5.7 × 10^−5^ a
0.5% NA	38.9 ± 4.7 a	22.8 ± 5.0 a	5475 ± 467 b	0.0015 ± 1.4 × 10^−4^ b
0.5% NS	38.2 ± 1.1 a	18.6 ± 2.7 a	4634 ± 255 ab	0.0015 ± 1.5 × 10^−4^ b
0.5% Kaolin	33.9 ± 2.6 a	17.9 ± 1.2 a	4031 ± 497 a	0.0014 ± 9.8 × 10^−5^ b

* Standard deviation. Averages followed by the same letter in the column do not differ significantly (Tukey test, *p* > 0.05).

## Data Availability

The original contributions presented in this study are included in the article. Further inquiries can be directed to the corresponding author.
